# 1-(2-Fluoro­benzyl­ideneamino)pyridinium bis­(1,2-dicyano­ethene-1,2-dithiol­ato)nickelate(II)

**DOI:** 10.1107/S1600536809015360

**Published:** 2009-04-30

**Authors:** Hui Zhang, Quan Zhou, Fengkai Hu, Han Xu, Guanru Chang

**Affiliations:** a Department of Chemistry, Huangshan University, Huangshang 245001, People’s Republic of China

## Abstract

In the title complex, (C_12_H_10_FN_2_)_2_[Ni(C_4_N_2_S_2_)_2_], the anion lies on an inversion center with the Ni^II^ ion coordinated by four S atoms in a slightly distorted square-planar environment. In the unique cation, the dihedral angle between the benzene and pyridine rings is 7.1 (2) Å.

## Related literature

For metal–[dithiol­ene]_2_ complexes, see: Ni *et al.* (2004 ▶, 2005 ▶); Nishijo *et al.* (2000 ▶); Ren *et al.* (2004 ▶); Robertson & Cronin (2002 ▶).
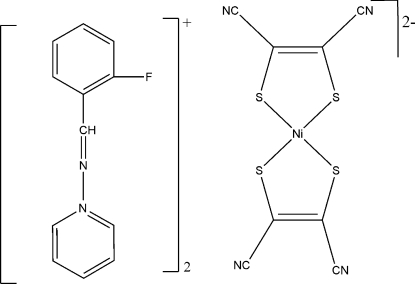


## Experimental

### 

#### Crystal data

(C_12_H_10_FN_2_)_2_[Ni(C_4_N_2_S_2_)_2_]
*M* *_r_* = 741.51
Triclinic, 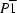

*a* = 7.9248 (13) Å
*b* = 9.1774 (15) Å
*c* = 11.1526 (18) Å
α = 88.326 (3)°
β = 77.202 (4)°
γ = 85.448 (4)°
*V* = 788.4 (2) Å^3^
*Z* = 1
Mo *K*α radiation
μ = 0.93 mm^−1^
*T* = 293 K
0.30 × 0.20 × 0.20 mm

#### Data collection

Bruker SMART CCD area-detector diffractometer
Absorption correction: multi-scan (*SADABS*; Bruker, 2000 ▶) *T*_min_ = 0.732, *T*_max_ = 0.809
4282 measured reflections
3030 independent reflections
1785 reflections with *I* > 2σ(*I*)
*R*_int_ = 0.038

#### Refinement

*R*[*F* ^2^ > 2σ(*F* ^2^)] = 0.052
*wR*(*F* ^2^) = 0.094
*S* = 0.76
3030 reflections
214 parameters
H-atom parameters constrained
Δρ_max_ = 0.31 e Å^−3^
Δρ_min_ = −0.24 e Å^−3^

### 

Data collection: *SMART* (Bruker, 2000 ▶); cell refinement: *SAINT* (Bruker, 2000 ▶); data reduction: *SAINT*; program(s) used to solve structure: *SHELXTL* (Sheldrick, 2008 ▶); program(s) used to refine structure: *SHELXTL*; molecular graphics: *SHELXTL*; software used to prepare material for publication: *SHELXTL*.

## Supplementary Material

Crystal structure: contains datablocks global, I. DOI: 10.1107/S1600536809015360/lh2806sup1.cif

Structure factors: contains datablocks I. DOI: 10.1107/S1600536809015360/lh2806Isup2.hkl

Additional supplementary materials:  crystallographic information; 3D view; checkCIF report

## Figures and Tables

**Fig. 1.**
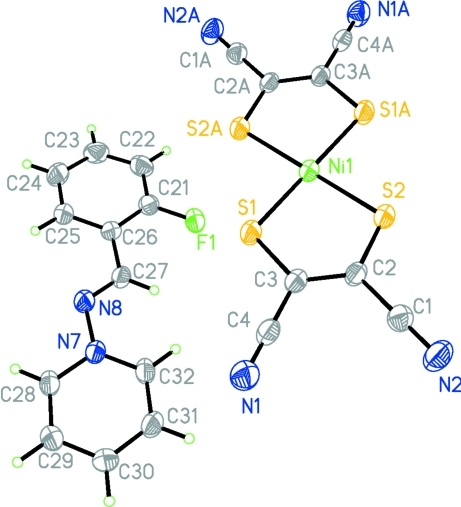
The molecular structure of (I), showing the atom-numbering scheme and displacement ellipsoids at the 30% probability level. Only the symmetry unique anion is shown. Symmetry code (A): -x, -y+1, -z.

## supplementary crystallographic information

### Comment

Molecular solids based on transition metal dithiolene complexes have attracted intense interest in recent years, not only owing to the fundamental research of magnetic interactions and magneto-structural correlations but also to the development of new functional molecule-based materials (Robertson & Cronin (2002). Much work has been performed on molecular solids based on *M*[dithiolene]_2_ complexes owing to their application as building blocks in molecular-based materials showing magnetic, superconducting, and optical properties (Nishijo *et al.*, 2000; Ni *et al.*, 2005). Herein we report the crystal structure of the title compound (I).

The molecular structure of (I) is illustrated in Fig. 1. The asymmetric unit contains one half [Ni(mnt)_2_]^2-^ (mnt = maleonitriledithiolato) dianion and one *o*-fluorbenzylidene-1-aminopyrazine cation, the formula unit being generated by an inversion center. In the [Ni(mnt)_2_]^2-^ cation the bond lengths and angles are in good agreement with related [Ni(mnt)_2_]^2-^ compounds (Ni *et al.*, 2004; Ren *et al.*, 2004)

### Experimental

Disodium maleonitriledithiolate (456 mg, 2.5 mmol) and nickel chloride hexahydrate (297 mg, 1.25 mmol) were mixed under stirring in water (20 mL) at room temperature. A solution of *o*-fluorbenzylidene-1-aminopyridinium bromide(665 mg, 2.5 mmol) in methanol (10 mL) was added to the mixture, and the red precipitate that was immediately formed was filtered off, and washed with methanol. The crude product was recrystallized in acetone (20 mL) to give red block crystals. Anal. Calcd. for C32 H20 F2 N8 Ni S4: C, 51.83; H, 2.72; N, 15.11%. Found: C, 51.96; H, 2.93; N, 15.03%.

### Refinement

The H atoms were placed in geometrically idealized positions (C—H = 0.93 Å) and refined as riding atoms, with *U*_iso_(H) = 1.2*U*_eq_(C).

### Crystal data

**Table:**

(C_12_H_10_FN_2_)_2_[Ni(C_4_N_2_S_2_)_2_]	*V* = 788.4 (2) Å^3^
*M**_r_* = 741.51	*Z* = 1
Triclinic, *P*1	*F*(000) = 378
Hall symbol: -P 1	*D*_x_ = 1.562 Mg m^−^^3^
*a* = 7.9248 (13) Å	Mo *K*α radiation, λ = 0.71073 Å
*b* = 9.1774 (15) Å	θ = 1.9–26.0°
*c* = 11.1526 (18) Å	µ = 0.93 mm^−^^1^
α = 88.326 (3)°	*T* = 293 K
β = 77.202 (4)°	Block, red
γ = 85.448 (4)°	0.30 × 0.20 × 0.20 mm

### Data collection

**Table:**

Bruker SMART CCD area-detector diffractometer	3030 independent reflections
Radiation source: fine-focus sealed tube	1785 reflections with *I* > 2σ(*I*)
graphite	*R*_int_ = 0.038
φ and ω scans	θ_max_ = 26.0°, θ_min_ = 1.9°
Absorption correction: multi-scan (*SADABS*; Bruker, 2000)	*h* = −8→9
*T*_min_ = 0.732, *T*_max_ = 0.809	*k* = −11→11
4282 measured reflections	*l* = −13→12

### Refinement

**Table:**

Refinement on *F*^2^	Primary atom site location: structure-invariant direct methods
Least-squares matrix: full	Secondary atom site location: difference Fourier map
*R*[*F*^2^ > 2σ(*F*^2^)] = 0.052	Hydrogen site location: inferred from neighbouring sites
*wR*(*F*^2^) = 0.094	H-atom parameters constrained
*S* = 0.76	*w* = 1/[σ^2^(*F*_o_^2^) + (0.0323*P*)^2^] where *P* = (*F*_o_^2^ + 2*F*_c_^2^)/3
3030 reflections	(Δ/σ)_max_ = 0.004
214 parameters	Δρ_max_ = 0.31 e Å^−^^3^
0 restraints	Δρ_min_ = −0.24 e Å^−^^3^

### Special details

**Table:**

Geometry. All e.s.d.'s (except the e.s.d. in the dihedral angle between two l.s. planes) are estimated using the full covariance matrix. The cell e.s.d.'s are taken into account individually in the estimation of e.s.d.'s in distances, angles and torsion angles; correlations between e.s.d.'s in cell parameters are only used when they are defined by crystal symmetry. An approximate (isotropic) treatment of cell e.s.d.'s is used for estimating e.s.d.'s involving l.s. planes.
Refinement. Refinement of *F*^2^ against ALL reflections. The weighted *R*-factor *wR* and goodness of fit *S* are based on *F*^2^, conventional *R*-factors *R* are based on *F*, with *F* set to zero for negative *F*^2^. The threshold expression of *F*^2^ > σ(*F*^2^) is used only for calculating *R*-factors(gt) *etc*. and is not relevant to the choice of reflections for refinement. *R*-factors based on *F*^2^ are statistically about twice as large as those based on *F*, and *R*- factors based on ALL data will be even larger.

### Fractional atomic coordinates and isotropic or equivalent isotropic displacement parameters (Å^2^)

**Table:**

	*x*	*y*	*z*	*U*_iso_*/*U*_eq_	
F1	0.5021 (3)	0.3555 (2)	−0.1896 (2)	0.0724 (7)	
N7	0.7362 (4)	0.1871 (3)	0.1593 (3)	0.0528 (8)	
N8	0.7118 (5)	0.1192 (3)	0.0532 (3)	0.0646 (10)	
C21	0.5600 (5)	0.2153 (4)	−0.2199 (4)	0.0561 (11)	
C22	0.5388 (6)	0.1628 (5)	−0.3283 (4)	0.0735 (13)	
H22A	0.4832	0.2200	−0.3804	0.088*	
C23	0.6029 (6)	0.0220 (5)	−0.3571 (4)	0.0797 (14)	
H23A	0.5949	−0.0156	−0.4320	0.096*	
C24	0.6779 (6)	−0.0643 (5)	−0.2792 (4)	0.0742 (14)	
H24A	0.7179	−0.1602	−0.3001	0.089*	
C25	0.6944 (5)	−0.0094 (4)	−0.1700 (4)	0.0609 (11)	
H25A	0.7452	−0.0686	−0.1166	0.073*	
C26	0.6362 (5)	0.1333 (4)	−0.1381 (3)	0.0484 (10)	
C27	0.6580 (5)	0.1978 (4)	−0.0257 (4)	0.0531 (10)	
H27A	0.6319	0.2974	−0.0126	0.064*	
C28	0.8296 (6)	0.1020 (4)	0.2236 (4)	0.0616 (12)	
H28A	0.8723	0.0089	0.1956	0.074*	
C29	0.8626 (6)	0.1496 (4)	0.3287 (4)	0.0704 (13)	
H29A	0.9291	0.0905	0.3725	0.085*	
C30	0.7965 (5)	0.2868 (4)	0.3703 (4)	0.0622 (12)	
H30A	0.8182	0.3215	0.4425	0.075*	
C31	0.6997 (6)	0.3708 (4)	0.3049 (4)	0.0667 (13)	
H31A	0.6533	0.4632	0.3326	0.080*	
C32	0.6708 (6)	0.3200 (4)	0.1996 (4)	0.0700 (13)	
H32A	0.6048	0.3780	0.1546	0.084*	
Ni1	0.0000	0.5000	0.0000	0.0484 (2)	
S1	0.17387 (15)	0.40603 (10)	0.11191 (9)	0.0589 (3)	
S2	−0.08272 (14)	0.68427 (10)	0.12200 (9)	0.0585 (3)	
N1	0.2744 (5)	0.4501 (4)	0.4175 (3)	0.0771 (12)	
N2	−0.0466 (5)	0.8166 (4)	0.4231 (3)	0.0776 (12)	
C1	−0.0194 (5)	0.7374 (4)	0.3440 (4)	0.0571 (11)	
C2	0.0169 (5)	0.6389 (4)	0.2423 (3)	0.0490 (10)	
C3	0.1256 (5)	0.5188 (4)	0.2397 (3)	0.0501 (10)	
C4	0.2087 (6)	0.4806 (4)	0.3384 (4)	0.0553 (11)	

### Atomic displacement parameters (Å^2^)

**Table:**

	*U*^11^	*U*^22^	*U*^33^	*U*^12^	*U*^13^	*U*^23^
F1	0.0756 (18)	0.0615 (15)	0.0818 (18)	0.0122 (13)	−0.0271 (14)	−0.0013 (13)
N7	0.063 (2)	0.0415 (18)	0.054 (2)	0.0031 (17)	−0.0156 (18)	−0.0038 (16)
N8	0.088 (3)	0.050 (2)	0.062 (2)	0.0107 (19)	−0.033 (2)	−0.0115 (18)
C21	0.049 (3)	0.057 (3)	0.061 (3)	−0.001 (2)	−0.010 (2)	−0.006 (2)
C22	0.076 (4)	0.084 (3)	0.067 (3)	−0.005 (3)	−0.031 (3)	0.004 (3)
C23	0.089 (4)	0.091 (4)	0.066 (3)	−0.004 (3)	−0.027 (3)	−0.023 (3)
C24	0.090 (4)	0.062 (3)	0.072 (3)	0.006 (3)	−0.022 (3)	−0.021 (3)
C25	0.070 (3)	0.049 (2)	0.068 (3)	0.003 (2)	−0.026 (2)	−0.006 (2)
C26	0.048 (3)	0.050 (2)	0.048 (2)	−0.006 (2)	−0.010 (2)	−0.0039 (19)
C27	0.053 (3)	0.044 (2)	0.061 (3)	0.003 (2)	−0.011 (2)	−0.007 (2)
C28	0.078 (3)	0.046 (2)	0.062 (3)	0.010 (2)	−0.024 (2)	−0.001 (2)
C29	0.091 (4)	0.058 (3)	0.068 (3)	0.013 (3)	−0.036 (3)	−0.005 (2)
C30	0.072 (3)	0.064 (3)	0.053 (3)	−0.001 (2)	−0.020 (2)	−0.009 (2)
C31	0.089 (4)	0.050 (3)	0.060 (3)	0.014 (2)	−0.019 (3)	−0.016 (2)
C32	0.095 (4)	0.053 (3)	0.066 (3)	0.026 (2)	−0.035 (3)	−0.012 (2)
Ni1	0.0527 (5)	0.0408 (4)	0.0520 (5)	0.0016 (3)	−0.0130 (4)	−0.0090 (3)
S1	0.0704 (8)	0.0504 (6)	0.0575 (7)	0.0119 (5)	−0.0214 (6)	−0.0151 (5)
S2	0.0655 (8)	0.0496 (6)	0.0631 (7)	0.0100 (5)	−0.0230 (6)	−0.0163 (5)
N1	0.100 (3)	0.068 (2)	0.068 (3)	0.008 (2)	−0.032 (2)	−0.010 (2)
N2	0.109 (3)	0.061 (2)	0.066 (3)	0.007 (2)	−0.028 (2)	−0.0186 (19)
C1	0.064 (3)	0.051 (3)	0.058 (3)	−0.002 (2)	−0.018 (2)	0.000 (2)
C2	0.050 (3)	0.039 (2)	0.056 (3)	−0.0034 (19)	−0.008 (2)	−0.0112 (19)
C3	0.057 (3)	0.043 (2)	0.051 (3)	0.000 (2)	−0.013 (2)	−0.0091 (19)
C4	0.068 (3)	0.042 (2)	0.055 (3)	0.001 (2)	−0.011 (2)	−0.011 (2)

### Geometric parameters (Å, °)

**Table:**

F1—C21	1.358 (4)	C29—C30	1.378 (5)
N7—C28	1.335 (4)	C29—H29A	0.9300
N7—C32	1.337 (4)	C30—C31	1.355 (5)
N7—N8	1.410 (4)	C30—H30A	0.9300
N8—C27	1.249 (4)	C31—C32	1.348 (5)
C21—C22	1.364 (5)	C31—H31A	0.9300
C21—C26	1.377 (5)	C32—H32A	0.9300
C22—C23	1.371 (5)	Ni1—S2^i^	2.1689 (10)
C22—H22A	0.9300	Ni1—S2	2.1689 (9)
C23—C24	1.360 (5)	Ni1—S1^i^	2.1703 (10)
C23—H23A	0.9300	Ni1—S1	2.1703 (10)
C24—C25	1.369 (4)	S1—C3	1.741 (3)
C24—H24A	0.9300	S2—C2	1.726 (4)
C25—C26	1.382 (5)	N1—C4	1.138 (4)
C25—H25A	0.9300	N2—C1	1.132 (4)
C26—C27	1.451 (4)	C1—C2	1.436 (5)
C27—H27A	0.9300	C2—C3	1.341 (5)
C28—C29	1.348 (5)	C3—C4	1.424 (5)
C28—H28A	0.9300		
			
C28—N7—C32	120.4 (3)	C28—C29—C30	119.2 (4)
C28—N7—N8	113.1 (3)	C28—C29—H29A	120.4
C32—N7—N8	126.4 (3)	C30—C29—H29A	120.4
C27—N8—N7	118.0 (3)	C31—C30—C29	119.3 (4)
F1—C21—C22	118.7 (4)	C31—C30—H30A	120.3
F1—C21—C26	117.7 (3)	C29—C30—H30A	120.3
C22—C21—C26	123.5 (4)	C32—C31—C30	119.8 (4)
C21—C22—C23	117.0 (4)	C32—C31—H31A	120.1
C21—C22—H22A	121.5	C30—C31—H31A	120.1
C23—C22—H22A	121.5	N7—C32—C31	120.6 (4)
C24—C23—C22	121.9 (4)	N7—C32—H32A	119.7
C24—C23—H23A	119.1	C31—C32—H32A	119.7
C22—C23—H23A	119.1	S2^i^—Ni1—S2	180.0
C23—C24—C25	119.7 (4)	S2^i^—Ni1—S1^i^	92.10 (4)
C23—C24—H24A	120.1	S2—Ni1—S1^i^	87.90 (4)
C25—C24—H24A	120.1	S2^i^—Ni1—S1	87.90 (4)
C24—C25—C26	120.7 (4)	S2—Ni1—S1	92.10 (4)
C24—C25—H25A	119.7	S1^i^—Ni1—S1	180.00 (4)
C26—C25—H25A	119.7	C3—S1—Ni1	102.69 (14)
C21—C26—C25	117.1 (4)	C2—S2—Ni1	102.72 (13)
C21—C26—C27	120.4 (4)	N2—C1—C2	178.9 (5)
C25—C26—C27	122.5 (4)	C3—C2—C1	121.6 (4)
N8—C27—C26	119.8 (3)	C3—C2—S2	121.5 (3)
N8—C27—H27A	120.1	C1—C2—S2	116.9 (3)
C26—C27—H27A	120.1	C2—C3—C4	121.9 (3)
N7—C28—C29	120.7 (4)	C2—C3—S1	120.3 (3)
N7—C28—H28A	119.7	C4—C3—S1	117.7 (3)
C29—C28—H28A	119.7	N1—C4—C3	179.7 (5)

Symmetry codes: (i) −*x*, −*y*+1, −*z*.
